# Real-Time Dynamic Imaging of Virus Distribution *In Vivo*

**DOI:** 10.1371/journal.pone.0017076

**Published:** 2011-02-15

**Authors:** Sean E. Hofherr, Kristen E. Adams, Christopher Y. Chen, Shannon May, Eric A. Weaver, Michael A. Barry

**Affiliations:** 1 Department of Laboratory Medicine and Pathology, Mayo Clinic, Rochester, Minnesota, United States of America; 2 Department of Internal Medicine, Translational Immunovirology and Biodefense Program, Division of Infectious Diseases, Mayo Clinic, Rochester, Minnesota, United States of America; 3 Department of Immunology, Department of Molecular Medicine, Mayo Graduate School, Mayo Clinic, Rochester, Minnesota, United States of America; 4 Institute for Molecular Medicine, University of Texas Health Science Center, Houston, Texas, United States of America; Louisiana State University Health Sciences Center, United States of America

## Abstract

The distribution of viruses and gene therapy vectors is difficult to assess in a living organism. For instance, trafficking in murine models can usually only be assessed after sacrificing the animal for tissue sectioning or extraction. These assays are laborious requiring whole animal sectioning to ascertain tissue localization. They also obviate the ability to perform longitudinal or kinetic studies in one animal. To track viruses after systemic infection, we have labeled adenoviruses with a near-infrared (NIR) fluorophore and imaged these after intravenous injection in mice. Imaging was able to track and quantitate virus particles entering the jugular vein simultaneous with injection, appearing in the heart within 500 milliseconds, distributing in the bloodstream and throughout the animal within 7 seconds, and that the bulk of virus distribution was essentially complete within 3 minutes. These data provide the first *in vivo* real-time tracking of the rapid initial events of systemic virus infection.

## Introduction

The fields of gene therapy, virology, cell and molecular biology, and others have all benefited from the use of reporter genes that allow the tracking of protein expression both *in vitro* and *in vivo*. For instance, reporter transgene expression has been used to track viral infection and spread in laboratory animals [1], [2], [3]. However, many next-generation viral and non-viral therapies will require systemic delivery [4]. Vascular administration of a virus or vector is inefficient in that a significant proportion of virus infects non-target tissues or is consumed by immune cells (reviewed in [5]). It is critical to understand where and how fast vector particles are being sequestered or conversely how long they are staying in circulation.

Reporter proteins are typically fluorescent proteins or luminescent proteins. Fluorescent proteins, such as GFP, DsRed, CFP, and others emit light when excited by a specific wavelength of light. Chemiluminescent reporters like luciferase, catalyze the reaction on a substrate often supplied *in trans* leading to photon release. Reporter genes have also been inserted into the genomes of viruses to track infection patterns and tropism. *In vivo* detection of these reporter proteins relies on imaging with sensitive charge-coupled device (CCD) cameras which have been integrated into complete whole-animal imaging systems. The evolution of imaging technology combined with the identification of improved reporter proteins has allowed researchers to effectively track viral transgene expression *in vivo*, but has a limited capacity to provide information about early kinetics and tropism of viral vectors.

A major limitation of using reporter genes for viral vector tracking and tropism is that only those viruses or vectors that are successful are tracked. That is, only vectors that succeed in cell entry and genome delivery generate a signal. In contrast, all failed or lost virions are undetected. Since the vast majority of virus particles are likely to be adsorbed without productive infection of cells, reporter gene imaging gives a skewed report of virus or vector distribution that may mislead interpretation of the pharmacology of these agents. In addition, the time necessary for the reporter transgene to be expressed in detectable quantities can be anywhere from hours to days post-injection, at which time, most vector trafficking has already occurred.

An alternate method for observing virion kinetics would be to label the particles with a molecule suitable for imaging. Fluorescent molecules that have been either chemically coupled to or genetically engineered into the viral capsid are an obvious candidate. Unfortunately, animal tissues have very high autofluorescence in the UV and visible light ranges making it difficult to resolve signal from noise using popular fluorophores or fluorescent proteins. Since there is little biological autofluorescence in the near-infrared infrared spectrum, new near-infrared fluorescent proteins are being developed. However, these are not sufficiently sensitive to track virions rather than transgenes *in vivo*.

In this work, we have conjugated the near-infrared (NIR) small molecule fluorophore IR800 to adenovirus (Ad) to track its rapid and realtime distribution *in vivo* in mice. We demonstrate this method can track virus distribution within seconds of injection and can be used to monitor the effects of engineering to modify virus tropism.

## Materials and Methods

### Virus Preparations

All viruses were grown, purified, and stored in 0.5 M KPBS buffer as previously described [6]. Ad-GL is a first generation version of adenovirus serotype 5 (Ad5) containing GFP-Luciferase fusion protein as a transgene under the control of the cytomegalovirus (CMV) promoter.

### Virus Labeling

IR800-CW was purchased from Licor Biotechnology (Lincoln, Nebraska USA). The dye was reconstituted according to manufacturer's directions in anhydrous dimethyl-sulfoxide (DMSO from Sigma) at a concentration of 5 mg/100 µL. The replication defective Ad5 (Ad-GL) was labeled at two different concentrations (high-dye and low-dye). The high-dye Ad-GL conjugation used 100,000 dye molecules/viral particle and the low-dye Ad-GL was labeled at a concentration of 10,000 dye molecules/viral particle. The labeling reaction was carried out for one hour at room temperature. Tris buffer was added to a final concentration of 50 mM to inactivate the remaining dye. Excess unbound free dye was removed by two consecutive size-exclusion columns (Bio-Rad DG). To determine the effectiveness of the free dye removal strategy, pure dye was treated with 50 mM Tris buffer to inactivate all NHS groups and then filtered through two size exclusion columns. The eluate was imaged with the purified high-dye and low-dye labeled virus and compared to a standard curve of dye that had been serially diluted. The mock treated Ad-GL was prepared under the same conditions as the labeled virus but in the absence of dye. The final concentration of each virus was determined by absorbance at 260 nm, and unless otherwise stated was 1×10^12^ viral particles/ml. Each mouse was injected with 200 µl of sample.

### Mice

All animal work was conducted after institutional approval by the Mayo Clinic IACUC, approval number A10109, Animal Welfare Assurance number A329101. Mice were 6–8 week old female Crl:SKHI-hr (outbred) and were purchased from Charles River. Because tail vein injection outside of the imaging system prevented imaging directly during injection, some experiments used mice bearing jugular vein catheters (JVC) surgically implanted by Charles River prior to shipment. This allowed virus to be injected directly into the mice already in the imaging box and enabled imaging to commence simultaneous with the injection. The catheter is a silicone catheter with a length of 82–88 mm and has a void volume is 10–12 µl. The tip of the catheter is placed in the right atrium. Once received, the mice were quarantined for 72 hours prior use. Mice were also ordered with a vascular access penny (VAP) port (Access Technologies Skokie, IL USA) implanted by Charles River prior to shipment. The VAP has a silicone port body with a length of 0.625 inches and a height of 0.25 inches and was directly tied into the right atrium of the animals.

### Realtime NIR imaging of labeled virus

Catheterized mice were anesthetized by intraperitoneal injection of 100 µl ketamine/xylazine (ketamine: 27.77 mg/ml; xylazine: 1.11 mg/ml), placed into the imaging box, then injected via their catheter with 200 µl of tris buffer containing 2×10^11^ Ad-GL particles labeled under high- or low-dye conditions. An additional mouse was injected with 200 µl of dye in Tris buffer with fluorescence that was equivalent to that of the high-dye Ad. For movie collection, one mouse was imaged for each condition using the catheterized mice. Injections in catheterized mice were validated by injections of a higher number of 5 mice per group by tail vein for the single 24 hour time point.

During and after these injections, near-IR images were taken using a Photometrics (Tucson, AZ USA) Lumazone Imager. Exposures were 250 milliseconds for a single image. Where movies were assembled, each image was taken every 500 milliseconds for 30 minutes. Images were processed using the Lumazone Imaging Software. The various time points of the realtime videos were captured using Hyperionics (Murrysville, PA USA) Hypersnap 6.0.

### Quantitation of Regions of Interest

Liver signal was quantified by generating an elliptical region of interest (ROI 1) in the location of the liver. This ellipse (ROI 1) was a fixed size for all images. The mean light intensity was calculated for ROI 1 at each time point using the Lumazone Imaging Software. Due to slight positioning differences between mice in the imager the ellipse was centered in the liver area for each mouse (intermouse experiment), but the location was fixed for each time-point (intramouse experiment). To control for background, the mean intensity was calculated by the Lumazone Imaging Software by generating a small square region of interest (ROI 2) in the region of the hind limb of the mouse. Similar to the liver quantitation, ROI 2 was a fixed size for all images, for intermouse experiments ROI 2 was repositioned and centered in the hind limb due to positioning differences between mice, and for intramouse experiments ROI 2 was in a fixed position.

### Assessing viral activity following IR-800 labeling

Non-catheterized mice were anesthetized with ketamine/xylazine (see above) then injected via the tail vein with 200 µl of tris buffer containing 2×10^11^ viral particles of Ad-GL labeled under high- or low-dye conditions. Control mice were injected with the same amount of non-labeled Ad-GL. The following day, these mice were anesthetized and then injected intraperitoneally with 100 µl of d-luciferin (20 mg/ml in PBS) and imaged for luciferase expression. Exposures were one minute in length and processed without binning. Images were processed using the Lumazone software for pseudocoloring the differing light intensities. Light intensity was quantitated by summing the detected lumens within a rectangular region of interest encompassing the entire animal. After imaging, the mice were euthanized and necropsied. Livers, spleens, and kidneys were isolated and imaged for near-infrared fluorescence as described above. N = 5.

## Results

Adenovirus serotype 5 (Ad5) vector expressing the reporter GFP-Luc (Ad-GL) was labeled at varied ratios with the amine-reactive NIR fluorophore IR800. IR800 was selected as the probe, since its excitation/emission at 745/795 nm provides better signal to noise than green or far-red fluorophores [7]. 5×10^12^ Ad-GL virus particles (vp) were reacted with IR800-CW dye at two ratios; 10,000 dye molecules/virion (low-dye) and 100,000 dye molecules/virion (high-dye). Excess dye was removed using two 6,000 kDa size exclusion chromatography columns. As a negative control, the same size exclusion protocol was performed on dye only, resulting in an eluate with no detectable fluorescence. 10^11^ vp of the IR800-labeled viruses were imaged on a Lumazone imaging system. 10^11^ vp (1.66×10^−13^ moles) of Ad labeled under low and high conjugation conditions had equivalent fluorescence to 10 and 100 picomoles (10^−11^ and 10^−10^ moles) of IR800. Labeling with lower ratios of IR800 produced virions below the limits of detection for the Lumazone system.

**Figure 1:**
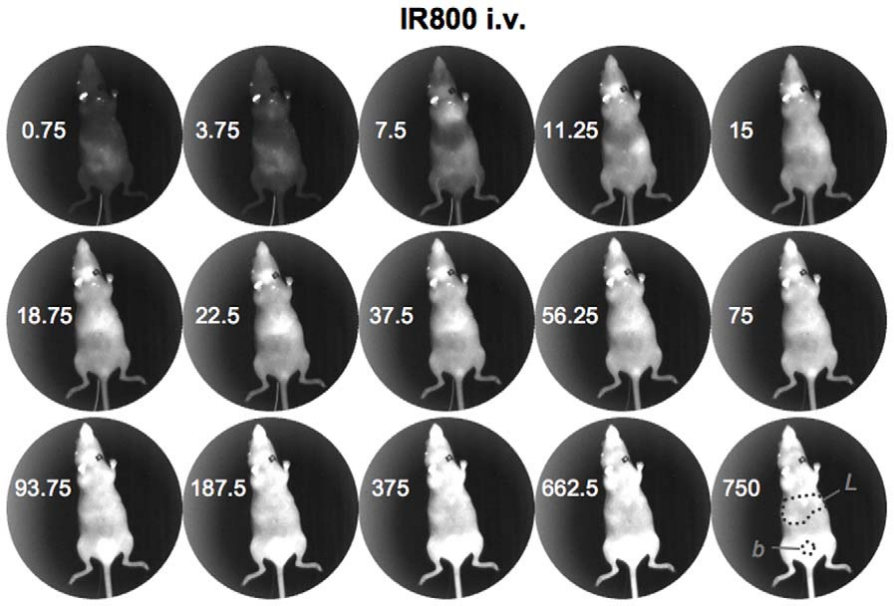
NIR imaging of IR800 free dye. Selected frames from a real-time image series taken of a catheterized mouse injected with near-infrared dye. Dark spot in the neck is from the catheter. Numbers indicate the time post-injection in seconds that the image was taken. “L” indicates the liver region (see also Figures 2 and 3 for liver localization of Ad-IR800). “b” indicates the bladder region where IR800 dye is eliminated from the body [7].

**Figure 2:**
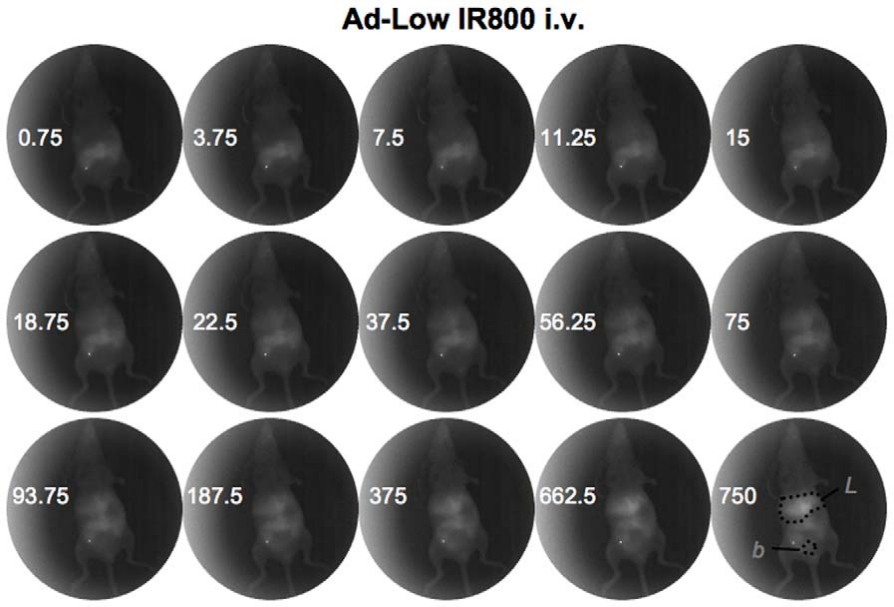
NIR imaging of Ad labeled with low IR800. Selected frames from a real-time image series taken of a catheterized mouse injected with Ad5 labeled with the low-dose of near-infrared dye. Dark spot in the neck is from the catheter. Numbers indicate the time in seconds post-injection that the image was taken.

**Figure 3:**
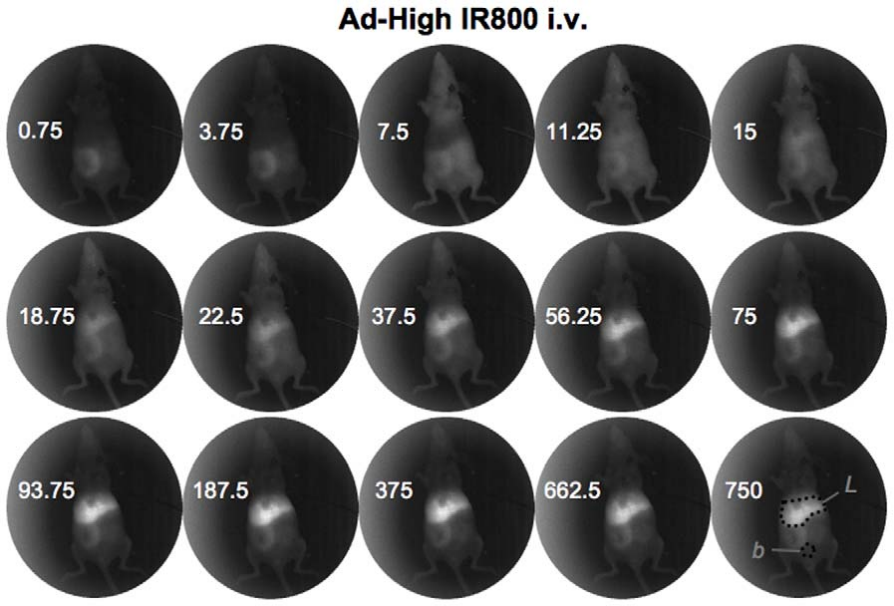
NIR imaging of Ad labeled with high IR800. Selected frames from a real-time image series taken of a catheterized mouse injected with Ad5 labeled with the high-dose of near-infrared dye. Dark spot in the neck is from the catheter. Numbers indicate the time in seconds post-injection that the image was taken.

Hairless mice were injected via a jugular catheter to allow imaging to occur essentially simultaneously with injection. Mice were injected with 2×10^11^ vp the IR800 labeled Ad-GL. For the low and high virus samples, this equated to injections of 20 and 200 picomoles of fluorophore per mouse. Dye control mice were injected with 200 picomoles of IR800, equal to the *in vitro* fluorescence of the high virus samples. The mice were imaged with the Lumazone in real time with 250 millisecond image captures taken every 500 milliseconds (see Figures 1, 2, and 3 for still individual images and see Supplemental Movies S1, S2 and S3 for realtime dynamic imaging).

Injection of free IR800 dye produced rapid distribution of the probe throughout the mouse within seconds of injection and this systemic fluorescence persisted through the end of the imaging time course. Although the entire mouse fluoresced, the most intense region of signal was in the bladder area (Figure 1 and Supplemental Movie S1). When either of the Ad samples (Ad-GL labeled with the low and high concentrations of IR800) were injected, a distinctly different distribution pattern was observed with highest signals from the virus labeled under the high conditions (Figure 2 and 3 and Supplemental Movies S2 and S3). Neither labeled virus sample (low nor high) demonstrated diffuse spreading through the injected animals, ultimately the signal localized predominantly to the liver.

**Figure 4:**
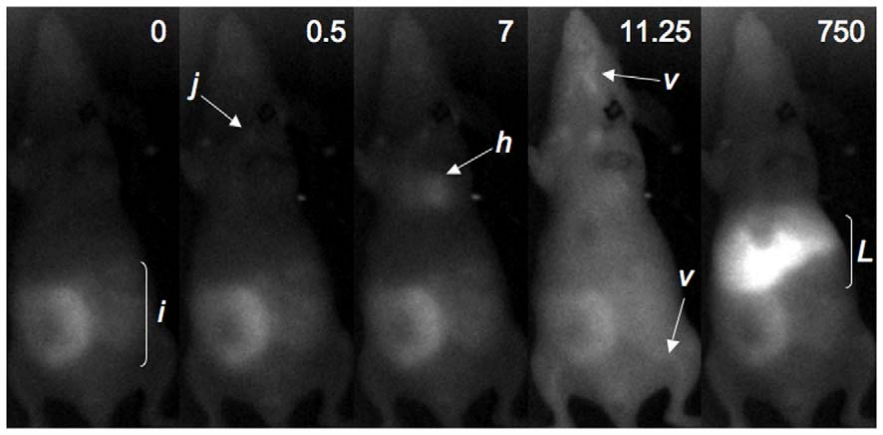
Enlargements of selected images from Figure 3 and Supplemental Movie S3, showing times and specific tissues that have high levels of infrared signal. i =  intestine; j =  jugular; h =  heart; v =  blood vessel; L =  liver.

**Figure 5:**
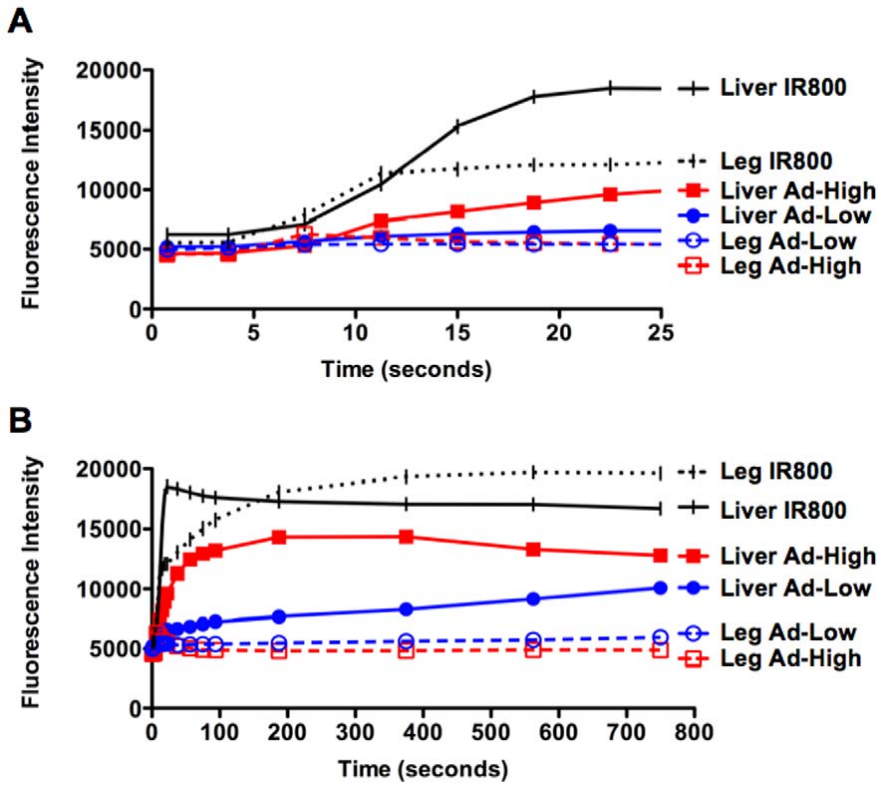
Quantitation of NIR fluorescence from liver and muscle for mice injected with dye alone or labeled viruses. Quantitation of NIR fluorescence from liver and muscle at early time points for a single mouse that was injected with dye alone or the indicated labeled virus. **A.** Early time points **B.** Later time points.

When images are analyzed more closely at each time point (Figure 4) and fluorescence distribution is quantitated (Figure 5), one can appreciate the rapid biodistribution of Ad virions after i.v. injection. At injection, background NIR fluorescence is observed only in the intestinal area due to autofluorescence of chlorophyll in the mouse chow. At injection, NIR fluorescence is observed entering the jugular. Within 500 milliseconds virus is detected in the heart. Within 7 seconds fluorescence is observed throughout the mouse with reduced signal in the liver due to its size and higher light absorbance. By 11.25 seconds, NIR fluorescence increases in the liver and can also be observed in the vasculature of the mice. Liver NIR signal increased at the expense of fluorescence elsewhere in the mice. This kinetic of signal loss is consistent with previous data studying the pharmacology of i.v. injected radiolabeled Ad5 [8]. By 187.5 seconds (3 minutes), liver NIR signal appears saturated for the high labeled Ad-GL group with slight reduction over 750 seconds (12 minutes) of imaging. Ad labeled at the lower level also accumulated in the liver, but with slower kinetics. This predominant liver signal is consistent with previous qPCR data demonstrating that approximately 95 to 99% of injected Ad5 is found in the liver within 30 minutes of injection [8].

Quantitation of NIR fluorescence in the liver and hind leg regions demonstrated distinctly different pharmacokinetics for free dye vs. labeled Ad (Figure 5). Free dye distributed rapidly to liver and leg with gradual loss in the liver and gradual increase in the leg. In contrast, liver signal for the IR800 labeled Ad-GL increased over time and leg signal was weak peaking at 7.5 seconds and then reducing to background. By 750 seconds, the NIR fluorescence ratio of liver:leg for free IR800 was 0.86 whereas this ratio was 3.08 and 1.59 for high and low labeled Ad-GL, respectively. This liver signal associated with IR800-labeled Ad-GL is consistent with previous work by tissue sectioning, enzyme assay, and reporter gene imaging that demonstrates that the liver is the predominant site of transduction by Ad5 after i.v. injection. Therefore, IR800 imaging of Ad5 virions grossly recapitulates the known tropism of this virus.

**Figure 6:**
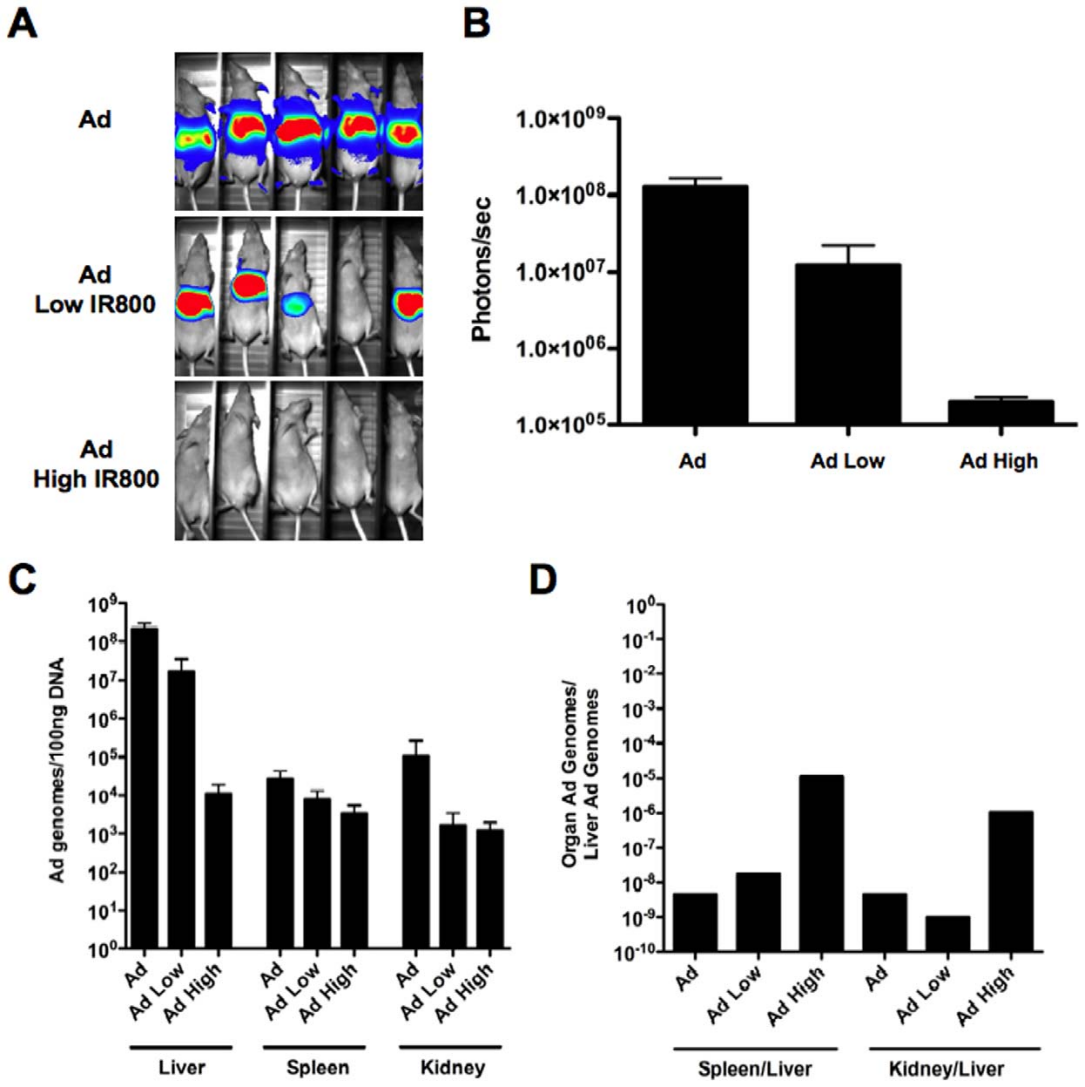
*In vivo* bioactivity and biodistribution of unlabeled and IR800-labed Ad-GL. Groups of 5 mice were injected by tail vein with unlabeled or low or high-dose labeled Ad-GL. 24 hours later, the mice were imaged for luciferase activity (**A** and **B**) and select organs were analyzed by qPCR for Ad genomes (**C** and **D**).

To determine the effect of labeling Ad5 with the 1.1 kDa IR800 molecule, groups of 5 hairless mice were injected i.v. by tail vein with the high-dye and low-dye labeled Ad-GL as well as a unlabeled Ad-GL. After 24 hours, luciferase transgene expression from the virus was measured by imaging to determine the effect of the labeling on virus activity (Figure 6A and B). Luciferase expression of the unlabeled Ad-GL injected mice was strong and restricted to the liver as expected from previous studies. Luciferase expression from the low-dye labeled Ad-GL injected mice was also seen in the liver, although the signal was ten-fold lower than unlabeled Ad-GL. In contrast, the high-dye labeled Ad-GL injected mice had detectable, but markedly lower luciferase expression.

**Figure 7:**
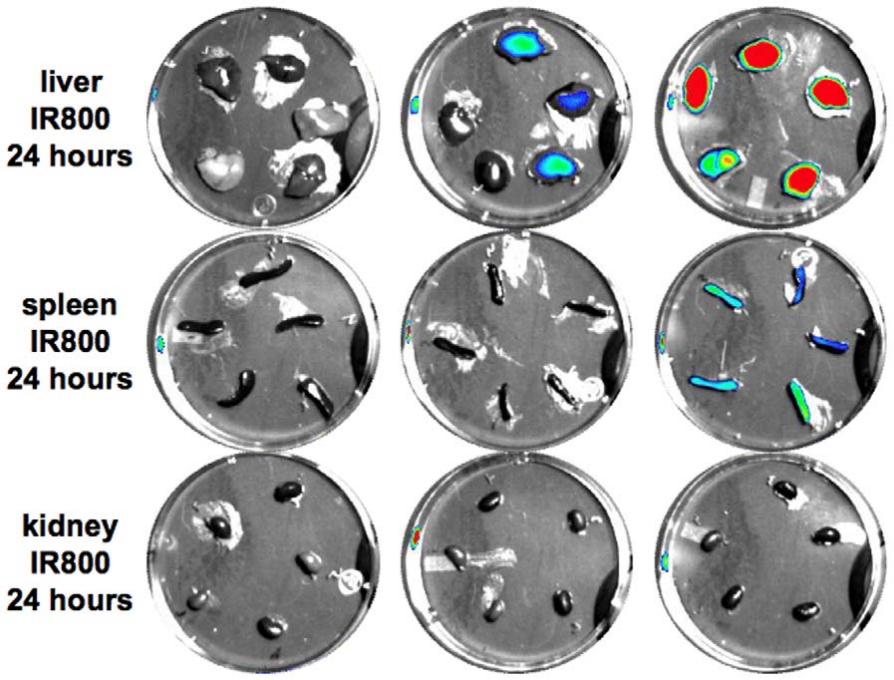
NIR fluorescence from harvested tissues from Figure 6. Organs that were harvested after injections were imaged for NIR fluorescence.

Organs were removed and imaged for NIR fluorescence (Figure 7). The livers and spleens of the high-dye labeled Ad-GL injected mice were all fluorescent. Three of five livers of the mice injected with the low-dye labeled Ad-GL had detectable fluorescence, but the intensity in this group was significantly lower than that of the high-dye group. None of the spleens from mice in the low-dye Ad-GL had overt fluorescence by imaging. No fluorescence was detected in the organs from the unlabeled Ad-GL injected mice.

Organs were also analyzed by quantitative PCR (qPCR) to measure the number of adenovirus genomes that were delivered (Fig. 6C and D). qPCR for unmodified Ad demonstrated highest viral genome load in the liver with similar lower levels in the kidney and spleen. The low and high-dye labeled groups showed reductions in Ad genomes in the liver that paralleled changes in luciferase activity with the low group having a modest reduction and the high group reduced 1000-fold. In the spleen, Ad genome levels were affected more modestly by IR800 modification of the virus. In the kidney, both labeled groups reduce viral genomes to similar levels.

These data indicated that the heavy dye labeling that best detects NIR fluorescence *in vivo* reduced, but did not ablate virus distribution in all organs. To assess to what degree labeling changed relative distribution, the spleen and kidney qPCR values were divided by those in the liver (Fig. 6D). This comparison indicated that low dye labeling did not drastically change the relative sites of virus distribution. However, the high labeling did change tissue distribution primarily by reducing viral genome content in the liver.

## Discussion

This work was initiated to monitor the immediate and realtime biodistribution of viruses in the seconds and minutes after i.v. injection, when reporter gene tracking is not feasible. We chose fluorophore labeling of virions as an approach that would be simple to apply in the laboratory and to avoid generating radioactive animals. Fluorescence imaging also has the advantage of more rapid image capture (milliseconds) than radioactive imaging methods. While this provides rapid kinetic imaging, fluorescence imaging is less effective at deep tissue imaging than SPECT or PET imaging, which have the added advantage of producing three-dimensional information [9], [10]. NIR fluorescence was used rather than fluorescence in the visible range, since wavelength in the 800 nm range have the lowest tissue absorbance and autofluorescence for dynamic imaging of pharmacokinetics as compared to imaging in the green range (530 nm) or red range (710 nm) [11]. While NIR is optimal, there is still some autofluorescence from the digestive tract of mice due to chlorophyll autofluorescence. The use of low autofluorescent chow can reduce, but not ablate this background to improve signal to noise ratios.

Two concentrations of IR800 dye per virion were used in this study: 100,000 dye molecules/virion and 10,000 dye molecules/virion. Labeling at lower ratios produced virus preparations that were below the sensitivity of detection by the current Lumazone NIR imaging system. When compared to standards of known dye concentrations, the 10,000 and 100,000 dye molecules per virion labeling conditions produced virions with fluorescence equivalent 60 and 600 IR800 molecules per virion. This does not mean there are these few fluorophores per virion, but is the fluorescence equivalent. In practical terms, these ratios are the highest that can be achieved to yield the most fluorescent viruses for detection with the current imaging system.

The low-dye labeled Ad-GL injected mice had weak signal, but the observed fluorescence was distinctly different from the dye alone. In these mice, signal traveled through the catheter and into the heart and thyroid regions of the animal. Over time, signal began to pool in the liver, this is concurrent with the known uptake of virus by Kupffer cells and blood factor mediated binding to hepatocytes [3], [12], [13], [14]. High-dye labeled Ad-GL followed the same kinetics but with higher signal intensity. These data are consistent with observations that more than 90% of Ad5 is being sequestered by the liver following i.v. injection.

The high labeling condition provided the most striking imaging quality particularly for kinetic tracking. Unfortunately, this level of labeling also skewed viral activity based on luciferase activity and qPCR of viral genomes. In contrast, the low labeling condition had modest effects on virus activity in the liver and only small effects on viral genomes in the tested organs which did not appear to have perturbed relative distribution. Given this, we anticipate that the use of NIR dyes with higher quantum yield and more sensitive imaging systems will allow labeling at the low dye and lower dye to virion ratio to enable imaging kinetics similar that observed for the high dye samples, but without perturbing function.

In summary, this is the first report of the real-time dynamic imaging at millisecond time scales of an intravenous infection by a virus. This technique should be valuable in monitoring viruses, viral and non-viral vectors, and nanoparticle distribution and pharmacokinetics in a qualitative and quantitative fashion. This proof of principle provides support for developing brighter fluorophores or more sensitive imaging systems to enable lower dye to virion ratios to be used.

## Supporting Information

Movie S1 **NIR imaging of IR800 free dye.** This movie corresponds to the still images of Figure 1.
(MOV)

Movie S2 **NIR imaging of Ad labeled with low IR800.** This movie corresponds to the still images of Figure 2.
(MOV)

Movie S3 **NIR imaging of Ad labeled with high IR800.** This movie corresponds to the still images of Figure 3.
(MOV)

## References

[1] Douglas JT, Rogers BE, Rosenfeld ME, Michael SI, Feng M (1996). Targeted gene delivery by tropism-modified adenoviral vectors.. Nat Biotechnol.

[2] Reynolds PN, Zinn KR, Gavrilyuk VD, Balyasnikova IV, Rogers BE (2000). A targetable, injectable adenoviral vector for selective gene delivery to pulmonary endothelium in vivo.. Mol Ther.

[3] Shashkova EV, Doronin K, Senac JS, Barry MA (2008). Macrophage depletion combined with anticoagulant therapy increases therapeutic window of systemic treatment with oncolytic adenovirus.. Cancer Res.

[4] Dubensky TW (2002). (Re-)Engineering tumor cell-selective replicating adenoviruses: a step in the right direction toward systemic therapy for metastatic disease.. Cancer Cell.

[5] Barry MA, Hofherr SE, Chen CY, Senac JS, Hillestad ML (2009). Systemic delivery of therapeutic viruses.. Curr Opin Mol Ther.

[6] Weaver EA, Nehete PN, Buchl SS, Senac JS, Palmer D (2009). Comparison of replication-competent, first generation, and helper-dependent adenoviral vaccines.. PLoS ONE.

[7] Adams KE, Ke S, Kwon S, Liang F, Fan Z (2007). Comparison of visible and near-infrared wavelength excitable fluorescent dyes for molecular imaging of cancer.. J Biomed Optics.

[8] Green NK, Herbert CW, Hale SJ, Hale AB, Mautner V (2004). Extended plasma circulation time and decreased toxicity of polymer-coated adenovirus.. Gene Ther.

[9] Ottobrini L, Ciana P, Moresco R, Lecchi M, Belloli S (2008). Development of a bicistronic vector for multimodality imaging of estrogen receptor activity in a breast cancer model: preliminary application.. Eur J Nucl Med Mol Imaging.

[10] Ray P, Wu AM, Gambhir SS (2003). Optical bioluminescence and positron emission tomography imaging of a novel fusion reporter gene in tumor xenografts of living mice.. Cancer Res.

[11] Adams KE, Ke S, Kwon S, Liang F, Fan Z (2007). Comparison of visible and near-infrared wavelength-excitable fluorescent dyes for molecular imaging of cancer.. J Biomed Opt.

[12] Lieber A, He CY, Meuse L, Schowalter D, Kirillova I (1997). The role of Kupffer cell activation and viral gene expression in early liver toxicity after infusion of recombinant adenovirus vectors.. J Virol.

[13] Shayakhmetov DM, Gaggar A, Ni S, Li ZY, Lieber A (2005). Adenovirus binding to blood factors results in liver cell infection and hepatotoxicity.. J Virol.

[14] Parker AL, Waddington SN, Nicol CG, Shayakhmetov DM, Buckley SM (2006). Multiple vitamin K-dependent coagulation zymogens promote adenovirus-mediated gene delivery to hepatocytes.. Blood.

